# COVID-19: The World Community Expects the World Health Organization to Play a Stronger Leadership and Coordination Role in Pandemics Control

**DOI:** 10.3389/fpubh.2020.00470

**Published:** 2020-09-08

**Authors:** Lidia Kuznetsova

**Affiliations:** Faculty of Medicine, Barcelona Institute for Global Health, University of Barcelona, Barcelona, Spain

**Keywords:** World Health Organization, COVID-19, international health regulations, pandemics, pandemic response, pandemic prevention

## Abstract

The coronavirus disease 2019 (COVID-19) pandemic has been accompanied by the return of the concept of national state and exhibited signs of crisis of globalism and liberalism. The pandemic affected most aspects of society and human activity, including socioeconomic impact. Economic problems, shortages of medical supplies and personnel, xenophobic sentiments, and misinformation led to the use of unethical practices and human rights violations. To navigate through this crisis, many countries resorted to traditional diplomacy in the absence of effective international instruments. Thus, the world faced the urgent need in functioning global governance. The pandemic also manifested the increasing importance of international organizations as sources of technical expertise, providing scientific basis for politicians to legitimize their decisions and actions. The article addresses the topic of implications of the pandemic for governance and forecasting a post-pandemic future. The research focus of this paper, therefore, is the assessment of the role of the World Health Organization (WHO) in prevention and response to pandemics. The work is aimed at identifying the functions of the WHO and assessing its activities in prevention and control of pandemics and response to the COVID-19 pandemic in particular. Furthermore, the objective of this article is to identify gaps in the WHO pandemic control efforts and formulate recommendations on addressing them.

## Introduction

The coronavirus disease 2019 (COVID-19) pandemic and other recent and ongoing infectious disease outbreaks, emerging, re-emerging, and neglected infectious diseases, as well as bioterrorism, posing a threat to health security, suggest the necessity and significance of pandemics-related research. The control of pandemics is impossible without international cooperation, due to their transboundary nature, and intergovernmental organizations are to play an important role in pandemic preparedness and response. The World Health Organization (WHO) is the only source of legally binding international regulations for pandemic response, the importance of which is growing, and a provider of technical assistance and standard guidelines to the states (1). Strong national health systems are the foundation for effective pandemics prevention and control, and their strengthening is crucial, especially in low-income countries. The international system of mechanisms of response to pandemics is currently in the process of formation, and it is a dynamic process. The challenge for such system is to ensure the existence of supranational legal authority and make it function. The authority and the capacity of the WHO to lead the international response have been questioned during the Ebola outbreak and the COVID-19 pandemic. The crises also revealed the lack of resources of the WHO to effectively prevent and respond to pandemics (2). At the same time, the role of emerging influential and resourceful actors in pandemic control has been growing, including the World Bank Group, the Bill and Melinda Gates Foundation, Médecins Sans Frontières, and other organizations. One of the central issues in international efforts to prevent and control pandemics is the aid to the poorest countries to develop health systems and ensure availability and accessibility to the basic health services by their population (3).

## The Role of the WHO in Pandemic Prevention and Control

The role of international mechanisms advanced significantly from adopting the WHO International Health Regulations (IHR) in 1969, focusing on just three diseases (cholera, plague, and yellow fever), to approving the current version of the IHR in 2005 and to creating the WHO Contingency Fund for Emergencies (CFE) in 2015 (4, 5).

During the SARS outbreak in 2003, the problem of coordinating response actions in different countries already became obvious. The existing response mechanisms were rather slow and disorganized. The outbreak revealed the necessity to modify the IHR. The revision of the IHR in 2005 allowed the WHO to declare Public Health Emergency of International Concern (PHEIC) and required the Member States to strengthen national emergency response capacity. The revised version of the IHR was tested by H1N1 influenza outbreak in 2009, when weaknesses in the global response to influenza pandemic were revealed again. The WHO issued recommendations to the Member States to create more extensive reserve global health workforce and establish $100 million contingency fund for future pandemics. However, these recommendations were not implemented until 2014 (6). The Ebola crisis revealed the importance of legal instruments and raised legal and ethical issues, due to, for example, introduction by some governments of trade and travel restrictions. This outbreak questioned the WHO credibility and the effectiveness of the IHR (7).

The WHO plays a key role among all intergovernmental organizations involved in tackling pandemics, and it is the only source of legal authority. The core functions of the WHO related to pandemics prevention and control include the following: support Member States in developing national capacity to respond to pandemics, support training programs, coordinate Member States for pandemic and seasonal influenza preparedness and response, develop guidelines, and strengthen biosafety and biosecurity (8).

The main instruments used by the WHO for pandemic prevention and control include the IHR, the Global Outbreak Alert and Response Network (GOARN), the Public Health Emergency Operations Centre Network (EOC-NET), the Contingency Fund for Emergencies, and the Pandemic Influenza Preparedness (PIP) Framework. At the strategic level in pandemic control, the WHO focuses on reinforcing national public health systems, One Health approach, and strengthening global partnership.

The IHR is a legally binding regime for protection and management of disease threats. It is a framework for collective response to the threats, involving one or more countries, or to public health events of global significance. The current version of the IHR entered into force on 15 June 2007, and they are binding on 196 countries across the globe, including all WHO Member States (1).

To date, the progress has been achieved in some areas of the IHR implementation, for example, introduction of national focal points to connect with different government sectors, stakeholders, and the WHO; increased transparency in reporting; improved use of early warning systems; and enhanced cooperation between organizations dealing with human and animal health. Nevertheless, there are still significant gaps related to the IHR. By the original deadline of June 2012, only one-fifth of the 192 WHO Member States had met the core capacity requirements, and by 2019, one-third (9). The problems related to the IHR implementation are lack of resources and difficulties in developing effective public health services. The IHR are not flexible enough to be adapted to local conditions. The criteria and mechanisms for declaring public health emergencies and for complying with the IHR need to be improved. The procedures should be simplified for the countries with scarce resources (3, 10).

In order to provide rapid access to resources and expertise for effective response to public health emergencies, in 2000, the WHO and partners established GOARN. The network provides a global operational framework encompassing a wide range of capacities and expertise, and it is aimed at coordinating support to countries and effectively deploying response teams. GOARN links over 200 institutions and networks and includes over 600 partners around the world (11). Since its establishing, the network has been involved in 135 field missions in 90 countries, deploying over 2900 professionals to the field (12). GOARN is considered to be effective, and it has gained trust and respect. The WHO stresses the importance of training and maintaining a reserve global health emergency personnel (13). GOARN focuses on the technical support roles and improving surveillance. Despite its efficiency, during Ebola outbreak, it became clear that GOARN needs to strengthen its leadership, respond faster, and broaden its capacity (6).

In 2012, the WHO established EOC-NET to identify and disseminate best practices and standards for EOCs and support EOCs' capacity building in Member States. The WHO works with EOC-NET partners to develop evidence-based guidance for establishing, operating, and improving EOCs (14).

Considering the criticism of the WHO in terms of lack of resources and slow response to emergency situations, CFE was established by the World Health Assembly in 2015, with the target funding of US$100 million for the 2018/2019 biennium. This target has been achieved. Since the establishment of CFE, the Member States have contributed over US$130 million to it (15). The distinctive feature of this fund is that it can be mobilized within 24 h, while the other financing mechanisms have different funding criteria and slower disbursement cycles. For this fund to be effective, it needs to attract greater levels of multi-year flexible financing (16).

PIP Framework for the sharing of influenza viruses and access to vaccines and other benefits is an international agreement adopted by the World Health Assembly in 2011 to improve global pandemic influenza preparedness and response. The Framework includes a PIP Benefit Sharing System that foresees an annual Partnership Contribution to the WHO from influenza vaccine, diagnostic, and pharmaceutical manufacturers through the WHO global Influenza Surveillance and Response System (17). Through this mechanism, the WHO will ensure the immediate availability of necessary products in case of influenza pandemic. Furthermore, WHO partners have contributed US$198 million to improve pandemic influenza preparedness and response. According to Gostin et al. (18), even though PIP Framework is not a treaty, it has features of international law, such as collective accountabilities, partners collaboration, and compliance procedures.

Global partnership is one of the main areas of work to guide the IHR implementation. Key partners to support the WHO implementation include the Food and Agriculture Organization, the World Organization for Animal Health, the UN Children's Fund, the International Labour Organization, the European Union (EU), international aid agencies, WHO collaborating centers, and non-governmental organizations and foundations (19).

## Response of the WHO to the COVID-19 Pandemic

According to the provisions of the IHR, on 30 January 2020, the WHO declared the outbreak a PHEIC and assessed the risk as very high for China, and high at the global level. On 11 March, the WHO said that the outbreak can be characterized as a pandemic (20). The WHO did not recommend limiting trade and movement, in line with IHRs. Many countries, however, have not followed these recommendations (21).

Shortly after announcing the pandemic, the WHO launched the COVID-19 Solidarity Response Fund. This initiative allows individuals and organizations around the world to directly support the work of WHO and partners to help countries with greatest needs prevent, detect, and respond to the COVID-19 pandemic. The disbursement mechanism for money raised through the Fund is quick and flexible. As of July 2020, the Solidarity Response Fund collected more than 200 million USD from more than 500,000 individuals and organizations (22). Furthermore, the WHO has also been involved in other fundraising efforts, such as establishing the WHO foundation and organizing charity concerts.

Another key initiative to respond to drastic medical supply shortages and potential food crisis in a number of countries, the WHO in collaboration with the World Food Program established the UN COVID-19 Supply Task Force in April 2020, within the framework of COVID-19 Supply Chain System. This mechanism has been created to coordinate the procurement of medical supplies to countries with overwhelmed health systems. This initiative will be run by the WHO and the World Food Program, together with a number of UN partners. The supply chain hubs will be located in Belgium, China, Ethiopia, Ghana, Malaysia, Panama, South Africa, and the United Arab Emirates. According to the WHO, the supply chain may need to cover more than 30% of the world's needs in the acute phase of the pandemic (23, 24). Prior to launching this mechanism, the WHO has already shipped personal protective equipment and diagnostic tests to over 120 countries.

The WHO has also launched a “Solidarity Trial” initiative, an international clinical trial, with the participation of 90 countries, aimed at finding effective treatment through rapidly discovering whether any existing drugs can slow the progression of the disease, or improve survival (25).

In collaboration with partners, the WHO launched a Global Collaboration to Accelerate the Development, Production, and Equitable Access to New COVID-19 diagnostics, therapeutics, and vaccines (26). The WHO has been extensively involved in providing training and technical assistance thought its OpenWHO platform and GOARN knowledge hub and in deploying experts via GOARN network (27). The WHO tackles misinformation through carrying out various online campaigns and being active on all social media channels. It releases daily situation reports and holds press conferences for updating the media about the pandemic. In March 2020, the WHO has started allocating the funds from CFE by releasing $9 million to the most vulnerable countries (28).

The response initiatives by the WHO have come under criticism, mainly by the US President Donald Trump, who accused the WHO for failure to control the pandemic and for promoting the interests of China. In April 2020, D. Trump announced the suspension of the US financing of the WHO and later on the withdrawal of the US membership in the WHO. However, other members such as China, France, and Germany pledged extra funding to the WHO to compensate for the lack of resources (29, 30). Thus, the WHO has been engaged in political confrontation, which has led to changes in balance and redistribution of influence among the Member States.

## Discussion

COVID-19 and previous pandemics have tested the leadership of the WHO and revealed a number of problems in its activities. The WHO response to both the 2009 influenza pandemic and the COVID-19 pandemic has been extensively criticized. The main points related to the WHO pandemic prevention and control activities that have come under criticism are as follows:

1. Over/underestimation of threat.
2. Conflict of interest and political bias.
3. Problems related to the IHR implementation.
4. Slow response.
5. Lack of financial resources.
6. The WHO is seen as a more political and less technical organization (6).
7. The WHO pandemic preparedness plans are ill-equipped to foresee and solve unique ethical challenges that may arise during different infectious disease outbreaks (31).

Apparently, the allegations of overestimation of threat and accusations of conflict of interest following the 2009 influenza pandemic have led the WHO to be more cautious in its statements and in declaring PHEIC and pandemic. The WHO followed experts' advice to mobilize the wider national, regional, and international community at earlier stages of an outbreak prior to a declaration of a PHEIC (3).

The majority of countries do not meet the core capacity requirements for the implementation of the IHR (9). A number of provisions the IHR have been violated by countries during the COVID-19 pandemic, as it had already happened during the Ebola pandemic (32). There is no multilateral strategy or funding to address the problem of pandemic preparedness and developing capacities for implementation of the IHR in low-income countries (7). At the same time, progress has been achieved in such areas as surveillance and communication among stakeholders involved in pandemics control and organizations dealing with human and animal health.

Some experts argue that the IHR do not create international law that is binding on the participant countries, due to the implementation and compliance problems. In practice, the international community applies “soft law” that implies non-binding duty to collaborate with other countries and with the WHO with regard to infectious disease surveillance and control of outbreaks. Although such “soft law” is neither mandatory nor enforceable, it is powerful politically. The reasons for why this “law” is functioning are that contributing to and enhancing international collaboration in infectious disease response is in a country's self-interest and that the WHO managed to create a framework for international cooperation on infectious diseases that is able to withstand the increasing global threats posed by pathogens (33). Suthar et al. (34) consider sanctions and embargoes a viable alternative to the functioning IHR. While using such measures can be inevitable in certain situations, as practice shows, these instruments can be based on the principle of double standard and be used for political manipulation purposes.

The WHO has been working on adjusting its policies and activities according to identified gaps, for example, by establishing the CFE. Experts point out evident progress in the WHO response to the Ebola outbreak in Congo in 2018, compared to its response to the 2014 outbreak (32). During the COVID-19 pandemic, the role of the WHO as a source of information and knowledge dissemination organization turned out to be critical, due to uncertain rapidly evolving situation and a lack of data and scientific knowledge about the virus and the disease. Given the significant impact of misinformation on countries' pandemic control efforts, this function of the WHO is especially important in the countries with low trust in government.

The WHO pays special attention to developing collaboration with other organizations involved in pandemics preparedness, focusing on One Health Approach. During the COVID-19 pandemic, the WHO has been collaborating and coordinating response with a wide range of international organizations, including the World Bank Group, various UN agencies, Gavi, the Global Fund, the EU, etc. (35).

The recommendations to improve the WHO capacity to prevent and control pandemics are as follows:

1. Continue the ongoing reform of the WHO.
2. Member States should ensure stable financing for the organization.
3. The WHO should work on increasing its credibility, paying special attention to ensuring the organization's transparency, political and business neutrality, and adapting evidence-based decisions and policies.
4. The member states should develop political trust, and the organization should be unbiased, distance itself from politics, and focus on its technical functions.
5. Focus the international efforts to tackle pandemics on long-term development aid programs and projects.
6. Concentrate efforts on developing basic health infrastructure and strengthen health systems in countries most vulnerable to pandemics.
7. Further consider the options for the IHR enforcement mechanism and the IHR revision.
8. Create a coordinated, adequately funded global health initiative to deliver assistance to the vulnerable countries to build their capacities to implement the IHR.
9. The WHO should further collaborate with partners to resolve the issues, indirectly related to the WHO functions, that impede effective prevention and control of pandemics.

The most vulnerable countries to pandemics are conflict-affected countries (36). Therefore, a powerful instrument to prevent pandemics is the prevention of conflict escalation. The aid efforts, including the efforts to strengthen health systems, will be ineffective and inefficient as long as the governments are involved in conflicts in the pursuit of taking over natural resources and boosting the profits of military corporations. Furthermore, the countries–beneficiaries of development aid can critically perceive the contradiction between the negative effects of economic policies dictated by the donors and development aid initiatives aimed at mitigating various effects of such policies on society and health of the population (37). Such issues, however, do not fall under the direct responsibility of the WHO, and the WHO cannot be held accountable for these shortcomings.

In response to the COVID-19 pandemic, the WHO has been working in line with its core functions related to pandemic control. It has used some of the existing mechanisms for pandemic prevention and control and created new ones to respond to COVID-19. Overall, given the situation of uncertainty and lack of knowledge about COVID-19, the WHO has taken timely appropriate steps in the initial response to the pandemic. The measures adopted by the WHO lie within the scope of the organization and have been limited by its mandate and available resources. Lessons learned from COVID-19 pandemic response should be further analyzed, and the organization's emergency response mechanisms and capacity should be improved, as discussed above. Many experts agree on the necessity to provide the WHO with more resources and stable financing and extend its mandate (2, 3, 38). The world community expects the WHO to play a stronger leadership and coordination role.

## Data Availability Statement

The original contributions presented in the study are included in the article/supplementary material, further inquiries can be directed to the corresponding author/s.

## Author Contributions

The author confirms being the sole contributor of this work and has approved it for publication.

## Conflict of Interest

The author declares that the research was conducted in the absence of any commercial or financial relationships that could be construed as a potential conflict of interest.
